# Incorporating translational research with clinical research to increase effectiveness in healthcare for better health

**DOI:** 10.1186/2001-1326-3-20

**Published:** 2014-07-05

**Authors:** Estela S Estape, Mary Helen Mays, Rosanne Harrigan, Robert Mayberry

**Affiliations:** 1 School of Health Professions, University of Puerto Rico Medical Sciences Campus, P.O. Box 365067, San Juan 00936-5067, Puerto Rico; 2 Puerto Rico Clinical and Translational Research Consortium, University of Puerto Rico Medical Sciences Campus, P. O. Box 365067, San Juan 00936-5067, Puerto Rico; 3 Department of Complementary and Alternative Medicine, University of Hawaii, 651 Ilalo St, Honolulu, MEB, Hawaii; 4 Morehouse School of Medicine, 720 Westview Drive, Atlanta, SW 30310-1495, Georgia

**Keywords:** Translational research, Clinical research, Research workforce, Effectiveness, Healthcare disparities, Comparative effectiveness research, Community-based participatory research

## Abstract

**Background:**

The transfer of new scientific discoveries into healthcare interventions requires that basic and clinical researchers work together with health care providers to generate team science. These innovative models require translational teams, and need to extend beyond the academic environment. The future of translational science requires partnerships with the healthcare community as well as the broader, general community. This new integrated model of effective translational teams holds promise for addressing thorny and persistent health disparities, is consistent with the nation’s strategic priority of eliminating health disparities, and bodes well for increasing healthcare effectiveness aimed at better health for all.

**Discussion:**

As part of the 13th Research Centers in Minority Institutions (RCMI) International Symposium on Health Disparities, several senior academic leaders joined efforts to hold a workshop to discuss a model that considers the incorporation of two translational research strategies in research career development programs: Comparative effectiveness research (CER) and community-based participatory research (CBPR) for increasing healthcare effectiveness and eliminating healthcare disparities. Discussion included what issues may be most germane to the concept of a unified model for research workforce development through formal training and career development leading to increased effectiveness in healthcare for better health.

**Summary:**

We believe that there is a gap in knowledge and skills in formal research career development programs that will enable physicians, other clinicians, and basic scientists to actively participate in these two translational research strategies. The purpose of this paper is to share the outcomes of these discussions, and encourage further discussion and possible innovation in the formulation of a new model for translational research workforce development.

## Background

Clinical and translational research is essential to generate and test interventions to reduce health disparities; however, health disparities within the United States persist. In its 2012 report, *‘How Far Have We Come in Reducing Health Disparities?: Progress since 2000’*[1]*,* the Institute of Medicine (IOM) noted that health disparities persisted both across time as well as the lifespan. The IOM also emphasized the importance of the community’s voice in reducing health disparities.

Support for translational research has increased as many academic programs now grant degrees in clinical research requiring achievement of the new translational competencies defined by National Institutes of Health (NIH) for post-doctoral master level clinical research programs [2]. Collaboration with communities and healthcare systems has increased as well as the recruitment of basic and clinical researchers, healthcare providers, and health professionals. The bidirectional continuum that defines translational research from basic or bench science (T1), transitioning through clinical settings (T2), clinical practice T3) to community/population applications (T4) is well known [2]. There is a need to understand and support the development of a practice and community-based translational research workforce.

## Methods

The 13th Research Centers for Minority Institutions (RCMI) International Symposium on Health Disparities was held in San Juan, Puerto Rico during December 2012. As part of this meeting, the Multidisciplinary Training and Career Development Key Function (MTCD) of the Puerto Rico Clinical and Translational Research Consortium (PRCTRC) and the Hispanic Clinical and Translational Research Education and Career Development (HCTRECD) programs collaborated with other academic leaders from minority institutions within a workshop to explore: (1) the role of translational research in clinical research and healthcare, (2) determine if there is a need for a unified model for research workforce development, and (3) ascertain if this intervention would increase the effectiveness of health interventions and reduce health disparities?

Translational Research Workforce Development, Healthcare Disparities, and Health Disparities are the three critical elements that were generated. Each will be discussed in the next section. Comparative effectiveness research (CER) and community-based participatory research (CBPR) were selected as two translational research strategies that have the potential to help transform healthcare and reduce healthcare disparities.

Translational Research Workforce Development is the process of training a new generation of researchers [3]. Proficiency knowledge and skill in conducting translational research is needed to improve health outcomes and eliminate health disparities. The conduct of translational research requires that multiple disciplines and representatives from different sectors work together as teams [3]. Team science is expected to result in the faster transfer of knowledge and other results into health benefits, while decreasing the economic burden of health costs.

In translational research, there is an emphasis on decreasing the time it takes to translate discoveries to practice and healthcare interventions, eliminating the gap that exists between research and practice, while facilitating research studies that focus on clinical practice. Academicians have responded to the lack of qualified investigators with the development of a curriculum that complies with new scientific trends and government policies [4]. These curricula require the skills and competencies that promote the dissemination and transfer of scientific advances, as well as the implementation of new paradigms for effective collaboration and resource sharing.

### Healthcare disparities

The 2002 Institute of Medicine (IOM) report, ‘Unequal Treatment: Confronting Racial and Ethnic Disparities in Health Care’ defined health disparities in healthcare as racial or ethnic differences in the quality of healthcare that were not due to access-related factors or clinical needs, preferences, and appropriateness of intervention [5]. The bases of these inequities included barriers within healthcare systems, complex issues related to patient-provider relationships, and individual challenges within patients and providers separately. Complex and bureaucratic systems, such as the US health care delivery system, are slow to change. The degree to which health disparities exist often reflect the choices made about the allocation of resources. As our nation struggles to reign in the cost of healthcare, health disparities have significant implications of not only the cost of inadequate care but the overall quality of healthcare in the US [6]. Research in health disparities must identify opportunities for appropriate interventions, particularly among groups with the greatest needs.

Comparative Effectiveness Research (CER) has been defined by the IOM as the “generation and synthesis of evidence” that compares the benefits and harms of alternative ways to improve care delivery or to “prevent, diagnose, treat, and monitor a clinical condition” [7]. In other words, by directly comparing existing health care interventions, we can determine which works best for which patients, and which poses the greatest benefits, and harms. The core question of CER is which treatment works best, for whom, and under what circumstances? The general importance of CER is reinforced by the evidence that it: (1) provides evidence to inform decisions and help improve health care; (2) helps decide what forms of healthcare interventions are best for a problem; and (3) evaluates which interventions are effective, on average, across a given patient population.

The inclusion of the community in equitable partnerships require sharing power, resources, credit, results and knowledge, as well as mutually appreciating the unique strengths that all parties bring to each stage of the project [8]. These partnerships, of which Community-based Participatory Research (CBPR) is an excellent example, should reflect equity in problem definition, research design, the actually conducting of the research project, interpreting the results, and determining how to use results for action. CBPR begins with a research topic of importance to the community and has the aim of combining knowledge with action and achieving social change [9]. CBPR differs from traditional research in several ways, the most striking being that it incorporates research, reflection, and action in a cyclical and iterative process. Unlike traditional ‘research’, CBPR most often seeks understanding through a cyclical process that combines research processes with reflection and action. Because CBPR focuses on issues that are of importance to the community, social inequities that often create and/or support health disparities and may serve as barriers to reducing disparities, are often brought to light during the process.

## Results and discussion

For each of these topics, several questions were developed and posed to the invited experts; their responses are based on their perspectives and experiences in clinical and translational research and translational workforce development.

### Research workforce development

**Figure 1:**
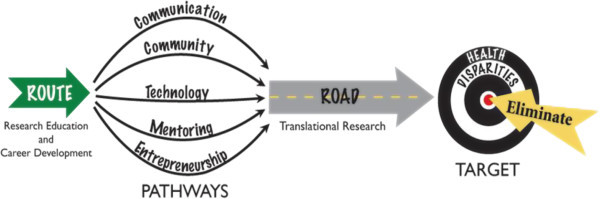
Translational research education pathways to eliminate health disparities.

*Which elements of translational research are the most relevant for advancing and improving healthcare practice?* Although the concept of translational research may have different meanings to each of us according to our own experience, it is important to note that it has a common goal: to accelerate the transfer of discovery to health benefit. For some, translational research is a term to express the integration of basic sciences and clinical research (“from bench to bedside”); for others, it means to accelerate the transfer of new knowledge to practice. Translational research has been defined in many ways, including fostering the multidirectional and multidisciplinary integration of basic research, patient-oriented research and population–based research; transforming scientific discoveries arising from laboratory, clinical or population studies into clinically relevant applications, and a process for developing evidence-based interventions and implementing them in practice [10]. As shown in Figure 1, some of the most relevant academic features needed for effective translational research education are: communication, community, technology, mentoring and entrepreneurship, and all have in common the goal of improving the health of the public.

Therefore, translational research can be advanced through many pathways leading to a process by which knowledge can travel at a faster pace to practice, thus ensuring that discoveries and innovations reach the patients or populations for whom they are intended. Taking this in consideration, it’s important to understand: *what are the major benefits of incorporating translational research with clinical research?*

Bringing multiple disciplines and different sectors with increasing diversity to work together as teams is expected to:

1. Facilitate a faster transfer of knowledge and scientific findings into health benefits.

2. Decrease the economic burden of health cost.

3. Eliminate the gap that exists between research and clinical practice.

4. Facilitate research studies that address the patient care problems encountered in common clinical practice.

Thus, we aim for a new health workforce that can integrate research efficacy with effectiveness. In order to achieve this aim, *what challenges in education have to be surpassed for an effective integration of translational and clinical research?* Many challenges have been identified, such as fragmented infrastructure, incompatible databases, regulatory burden, career disincentives, practice limitations and lack of funding. There is a strong movement both in the government and private sector to surpass these challenges. Academicians are working to respond to these challenges with the implementation of new paradigms for effective collaboration and resource sharing [11]. These translational competencies are best achieved in a multidisciplinary learning and working environment where leadership, critical thinking and networking skills are medullar in the education and career development of this new generation of translational researchers.

### Healthcare disparities

W*hy is it important to study healthcare disparities?* Ours is a society that continues to wrestle with a legacy of discrimination based on color, educational attainment, income, gender, ethnicity, and sexual orientation [6]. Facing past and present discrimination poses moral and ethical issues for healthcare providers and administrators, particularly in healthcare systems that have supported unequal distribution of resources. We are also asked to consider healthcare as a resource tied to social justice, opportunities, and the quality of life of individuals and groups (facilitating the advancement of persons economically and professionally).

*What are some of the contextual and explanatory factors of racial and ethnic healthcare disparities?* A review of racial and ethnic disparities in access to and use of medical care reveals clear and disturbing trends of significant differences in access to and use of medical care by race and ethnicity within certain disease categories and types of healthcare services [6]. Factors such as a patient’s socio-economic status, insurance coverage, health status, disease severity, availability of needed services, and patient preferences do not fully explain racial and ethnic differences in access to medical care. Data indicate that interpersonal factors (e.g., culture, provider bias, cultural perceptions and differences, discrimination, and intentional and unintentional bias) may explain some causes of healthcare disparities. Least is known about healthcare system-level characteristics, although these variables may offer some explanation.

**Figure 2:**
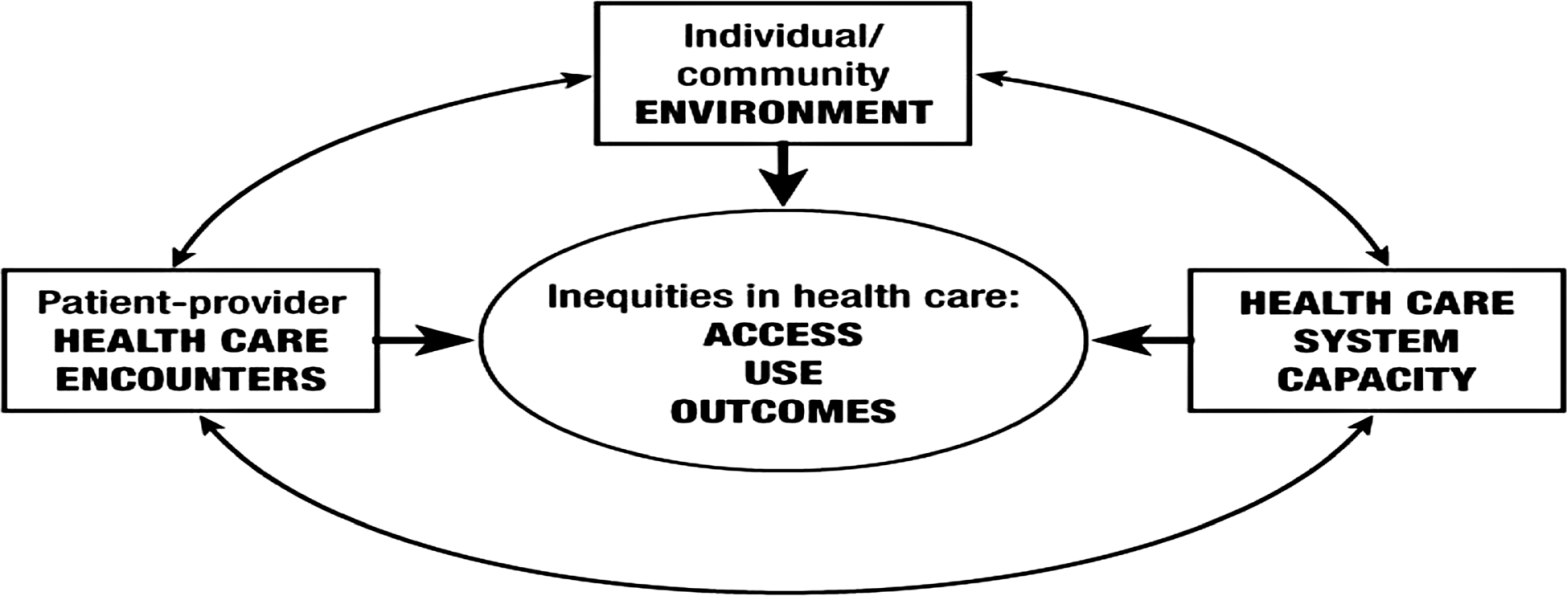
Theoretical model of health equity.

In order to more accurately identify and address those factors that contribute to healthcare disparities, we must ask *if we can describe some of the general approaches and strategies for eliminating healthcare disparities and achieving equitable healthcare*. One theoretical model of healthcare equity [12], as shown in Figure 2, would require nine factors for success:

1. Continue to increase awareness among providers and decision makers of the importance of equity in “achieving best care”.

2. Insure equitable access to preventive and curative health care services.

3. Reduce uncertainty in clinical decisions, through real-time patient medical record and clinical laboratory information, evidence-based guidelines and recommended standards of best care.

4. Improve efficiency and coordination of all aspects of primary care, ambulatory care, specialty care, and hospital care.

5. Eliminate variations in health care, e.g., due to unconscious and conscious cost considerations, reimbursements, and patient insurance coverage or perceived ability to pay.

6. Increase the knowledge base to better understand the nature and causes of health care inequities and appropriate interventions to eliminate them.

7. Create a culturally competent health care system capable of delivering the highest quality and safest care available to every patient regardless of race, ethnicity, social class, culture, ability to pay, or language proficiency.

8. Insure health system accountability in equitable care quality by tracking, monitoring and reporting equity quality measures.

9. Integrate eliminating inequities with quality improvement processes.

### Comparative effectiveness research

The core question of comparative effectiveness research (CER) *is which treatment works best, for which patients, and under what circumstances?* The American Recovery and Reinvestment Act of 2009 allocated $1.1 billion for CER [13], which seeks to inform healthcare decisions by examining evidence on the effectiveness, benefits, and harms of different treatment options. With this information, providers and patients are able to make more informed treatment decisions that improve health outcome, taking into account the patient’s preference, values, and experiences. Its focus on evidence-based comparisons of treatment options, together with the joint decision-making process of patient and provider, makes CER an effective approach to improving healthcare outcomes, but also in reducing healthcare costs and eliminating health disparities.

A factor for consideration in CER is that of treatment heterogeneity or the heterogeneity of treatment effect (HTE). Randomized clinical trials (RCTs), considered the gold standard in clinical research [14], may produce data indicating that the same treatment may have different levels of impact of different people although outcomes may be reported as an average treatment effect (ATE), implying that treatment outcomes may be similar across populations with shared (heterogeneous) characteristics. As CER seeks improved decision-making among patients and providers, clinicians’ consideration of evidence from RCTs would include information about treatment variations within the study populations.

A correlated concept to CER is that of patient-centered outcomes research (PCOR), which evaluates the outcomes of healthcare practices, identifying ways that patients may be more involved with their own healthcare and making informed decisions about their health outcomes [13]. In 2010, the Patient Protection and Affordable Care Act (PPACA) established the Patient-Centered Outcomes Research Institute (PCORI), a national program in CER.

Ultimately, CER and PCOR seek to address the complexity of how clinical treatment options are evaluated and applied in a multi-dimensional environment that represents a variety of stakeholders including patients, providers, healthcare organizations, and insurance companies, as well as healthcare policy makers. The future of healthcare and its innovations will require a well-trained workforce capable of conducting comparative effectiveness research which will in turn advance translational science.

### Community-Based Participatory Research

Community-based participatory research (CBPR) has been defined as research that is conducted as an equal partnership between traditionally trained “experts” and members of a community [15]. In CBPR projects, the project begins with the community and the community participates fully in all aspects of the research process. This collaborative approach to research equitably involves all parties in the research process, recognizing the unique strengths that each brings to the study. Researchers who choose to engage in CPBR should ask themselves, *‘Do you believe that attending to social inequities should be part of a research agenda?*’ Some researchers may be concerned that addressing social inequities may cloud the research process, thus reducing objectivity and the overall integrity of the research design. In fact, social inequities must be addressed as these situations where groups or ‘communities’ do not have equal social status, social class, and/or social circles may have created or contribute to a myriad of health disparities. Unaddressed, these divisions (which are often economically based), can lead to social divisions as well as perpetuating discrimination in key issues such as access to healthcare [6].

The very nature of the CBPR process requires an ecological perspective that examines determinants of health from more than one ecological level (e.g., individual, interpersonal, community, organization or policy). By definition, this would require a more complex research design requiring objectives at more than one ecological level. This characteristic of CBPR may require researchers to ask themselves, *‘Do you question the need to address health – and therefore your research – from an ecological perspective?’* The CBPR researcher must be prepared to understand and navigate the dynamic interrelationships between and among personal, environmental, social, and political factors that shape and define ‘community’.

Before entering the field of CBPR, researchers should ask themselves *‘Do you perceive community participation as exploitative rather than empowering?’* The definition and intent of community-based participatory research is often misinterpreted to be the same as ‘community-oriented’ or ‘community-focused’. These approaches are very different from CBPR as they do not assume a proactively planned partnership relationship with community in the research process. In these models, communities are often used as ‘labs’ where data are collected, analyzed, and reported. But, there is little change within the community’s health, social, or economic status at the end of a research project. Communities in general may be distrustful of ‘outside experts’. Those with negative experiences where they believe the community has been exploited by the ‘expert’ are much less likely to open themselves to any partnership with the research academy. It can be a burden for the researcher to assure the community that the CBPR process is not exploitative. And, these assurances must be validated by actions.

## Conclusions

A unified model of training the next generation of clinical and translational research should include: 1) a better understanding of health disparities and approaches to achieve health equity, 2) comparative effectiveness research training to better understand the relative benefits of alternative deliveries of healthcare, 3) community participation in all aspects of research and healthcare decision. What is the need for a unified model of training for this new workforce, and how would such a model for workforce development increase effectiveness in healthcare and reduce health disparities? A few suggested approaches to addressing the challenges of preparing this new generation of translational research workforce are:

1. *Proactive recruitment and inclusion of healthcare providers in existing academic programs that grant degrees in translational research*. Healthcare providers, by the nature of their work, are often unable to attend classes offered in traditional ‘brick and mortar’ environments. As a result, academic programs seeking greater participation of healthcare providers in their degree programs will need to develop more online offerings as well as other types of learning strategies such as team facilitation and field research projects that encourage the cooperative work between non-clinicians and clinicians as well featuring more experiential learning opportunities.

2. *Develop strategies for teaching how to effectively engage multiple stakeholders across a variety of organizations and environments*. Healthcare and community environments feature an array of stakeholders, each representing unique views, interests, and needs. Healthcare administrators’ interest in CER may be primarily linked to cost containment while providers’ interest is likely grounded in the effectiveness of treatment options and improved health outcomes. Both views are valid, given the stakeholders’ role in healthcare. When proposing a CER study, translational researchers will need to not only be aware of these differences, but understand how to work with each so that well-designed studies can be implemented while addressing each stakeholders needs.

3. *Develop collaborative relationships between translational researchers within the academy and stakeholders in healthcare and community*. Effective collaborations are those that reflect full-partnerships between the academy and the healthcare entity or community including a willingness to share decision-making authority, roles in the research study, and publication recognition. In the same way that translational researchers are discouraged from considering communities as ‘research labs’, the same philosophy applies to healthcare.

In conclusion, clinical and translational research is seen as a cornerstone in reducing health disparities, and increasing effectiveness in healthcare for better health. While the need for research as the T1 and T2 stages of the translational research continuum is not diminished, as noted by the IOM [1] clinical care within the healthcare system and the role of communities in reducing health disparities present clear ‘next steps’ in the efforts to reduce health disparities.

## Abbreviations

UPR-MSC: University of Puerto Rico, Medical Sciences Campus; CBPR: Community-based participatory research; CER: Comparative effectiveness research; US: United States.

## Competing interests

Authors have no competing interests, financially or non-financially, to declare.

## Authors’ contributions

EE developed the topic on the Research Workforce Development, RH developed the topic on Community-based Participatory Research, RM developed the topic of Health Disparities and MHM synthesized information related to this debate into the final manuscript. All authors read and approved the final manuscript.
